# Pseudoprogression of Non-Small Cell Lung Cancer Following Neoadjuvant Therapy: A Case Report

**DOI:** 10.7759/cureus.92448

**Published:** 2025-09-16

**Authors:** Kareem Ramadan, Mona Benaissa, Michael Shackcloth

**Affiliations:** 1 Cardiothoracic Surgery, Liverpool Heart and Chest Hospital, Liverpool, GBR; 2 Histopathology, Royal Liverpool University Hospitals, Liverpool, GBR

**Keywords:** lung adenocarcinoma, neoadjuvant therapy, nos, nsclc, radiological pseudo progression

## Abstract

Neoadjuvant chemoimmunotherapy is increasingly used in early-stage non-small cell lung cancer (NSCLC) and has demonstrated improved pathological response rates. However, radiological progression following treatment may result in potentially curative surgery being withheld.

We report the case of a 75-year-old male with a poorly differentiated adenocarcinoma of the right lower lobe lung adenocarcinoma treated with neoadjuvant carboplatin, paclitaxel, and nivolumab. Post-treatment CT scan showed radiological progression. The patient underwent video-assisted thoracoscopic right lower lobectomy with posterior segmentectomy. Histopathological examination revealed a complete pathological response with no viable tumor and no nodal involvement.

This case highlights the importance of recognizing pseudoprogression in the neoadjuvant setting.

## Introduction

Neoadjuvant chemoimmunotherapy has shown encouraging results in the treatment of early-stage non-small cell lung cancer (NSCLC) [1], leading to improved pathological response rates and event-free survival [2]. However, tumors that show radiological progression following neoadjuvant chemoimmunotherapy may be deemed not suitable for surgery.

Pseudoprogression is a phenomenon characterized by radiological evidence that suggests the tumor has progressed, when, in fact, the tumor has responded to treatment [3]. We present a case with radiological progression following neoadjuvant chemoimmunotherapy that had a complete pathological response (cPR) following surgery.

## Case presentation

Written informed consent was obtained from the patient to share the data for educational and scientific purposes.

**Figure 1:**
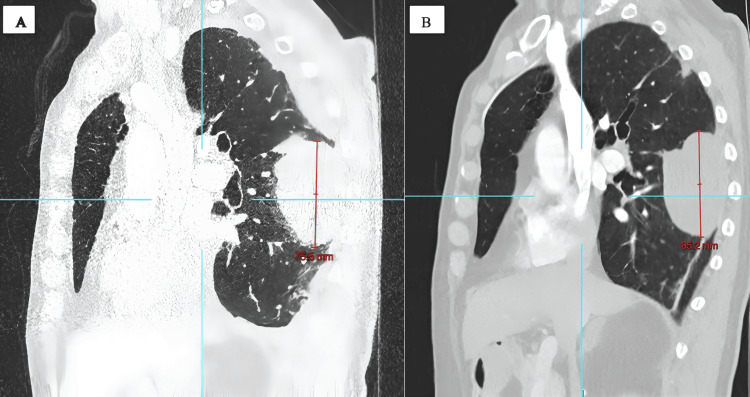
Sagittal CT chest image showing the size of the mass (A) before and (B) after neoadjuvant therapy.

**Figure 2:**
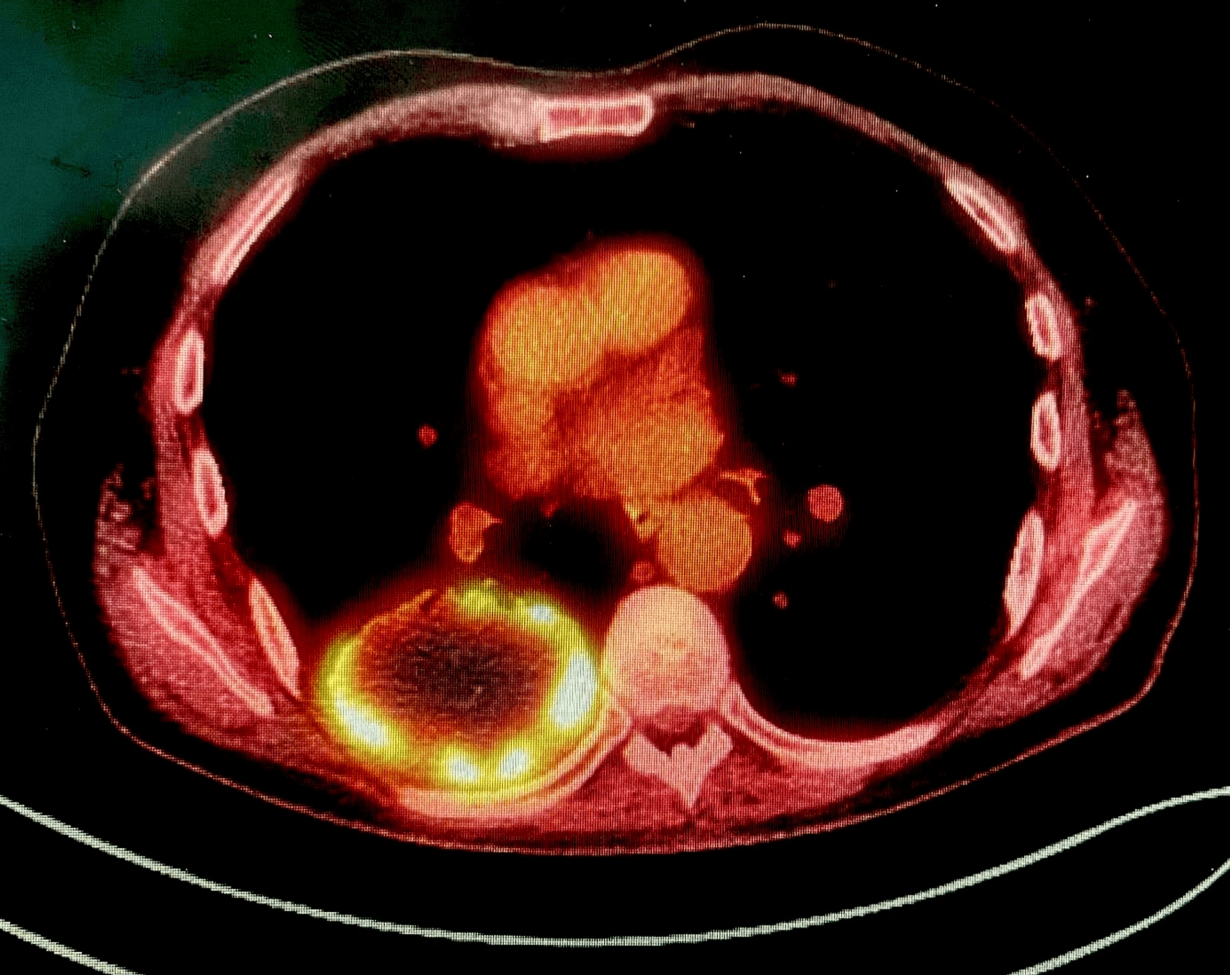
PET-CT chest shows a PET-avid mass. PET-CT, positron emission tomography-computed tomography

 A 75-year-old man presented with lower back pain and sciatica. Magnetic resonance imaging (MRI) of the spine was performed, which revealed a large mass in the right lower lobe. A computerized tomography (CT) scan of the chest (Figure 1A), abdomen, and pelvis was performed, which showed a 75 mm x 50 mm mass in the posterior aspect of the right lower lobe. Positron emission tomography-computed tomography (PET-CT) scan revealed high uptake (SUVmax 13.5) in the right lower lobe mass (Figure 2), with no significant lymph node uptake or distant metastasis. CT-guided biopsy revealed a non-small cell carcinoma, not otherwise specified (NOS), with no glandular or squamous differentiation, negative for epidermal growth factor receptor (EGFR) and anaplastic lymphoma kinase (ALK) mutations, and with a programmed death-ligand 1 (PD-L1) expression of 100%. The patient was deemed fit for multi-modality treatment and underwent three cycles of neoadjuvant chemoimmunotherapy with carboplatin, paclitaxel, and nivolumab.

A repeat staging CT scan (Figure 1B) demonstrated an increase in lesion size from 75.5 to 85 mm in maximal diameter. However, the tumor remained anatomically resectable. The patient underwent a right video-assisted thoracoscopic surgery (VATS) lower lobectomy with posterior segmentectomy of the right upper lobe, as the lesion crossed the fissure.

**Figure 3:**
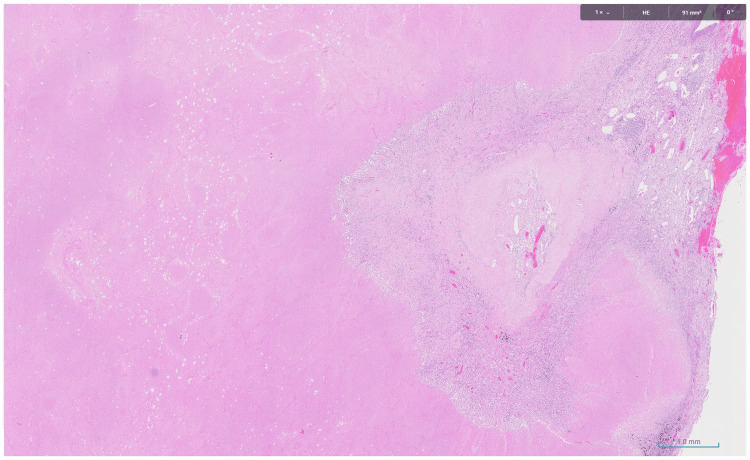
Focal stromal area with fibrosis, inflammation, and necrosis.

Postoperatively, the patient developed a chest infection, which led to a prolonged hospital stay of 15 days. The histopathology revealed no viable tumor, with 90% necrosis and 10% fibrotic stroma (Figure 3). All lymph nodes were negative for malignancy.

## Discussion

One limitation of neoadjuvant chemoimmunotherapy is that patients with resectable disease may not undergo surgery because of disease progression or treatment-related complications. In the CheckMate 816 study, 17% of patients did not undergo surgery, with 37% of these cases attributable to disease progression [4].

Pseudoprogression is the term used to describe an apparent radiological progression following immune checkpoint inhibitor therapy, after which subsequent imaging reveals tumor regression or stability. In a meta-analysis, Park et al. estimated the incidence following immunotherapy is around 6% in solid tumors [5-6]. In one of the early series of neoadjuvant chemoimmunotherapy, Forde et al. [6] described a case where radiological progression was observed despite downstaging at surgery. The underlying mechanism is thought to be immune-cell infiltration and an inflammatory reaction in the tumor microenvironment, causing radiological enlargement despite actual tumor necrosis [7,8].

This discordance between radiological and pathological response complicates clinical decision-making and may lead to patients being inappropriately excluded from surgery. In the NADIM (Neoadjuvant chemotherapy and nivolumab) trial [9], 33% of patients with radiologically stable disease had a cPR at surgery, underscoring the limitations of radiographic criteria when used in isolation.

Following neoadjuvant chemoimmunotherapy, we strongly recommend that patients not be denied treatment based on radiological progression alone. It is our institutional policy that if the tumor is still operable, then we would progress to surgery without further investigations. If new N3 lymph node involvement or evidence of metastatic disease is detected, biopsy confirmation is required before altering the surgical plan.

## Conclusions

This case highlights the importance of recognizing pseudoprogression in the neoadjuvant setting. We strongly advise that any radiographic progression following neoadjuvant treatment should be interpreted with caution, and patients should not be excluded from surgery unless there is histological evidence of nodal or distant metastatic progression.
